# Hypoxia-regulated gene expression explains differences between melanoma cell line-derived xenografts and patient-derived xenografts

**DOI:** 10.18632/oncotarget.8181

**Published:** 2016-03-18

**Authors:** Joydeep Bhadury, Berglind O. Einarsdottir, Agnieszka Podraza, Roger Olofsson Bagge, Ulrika Stierner, Lars Ny, Marcela Dávila López, Jonas A. Nilsson

**Affiliations:** ^1^ Department of Surgery, Institute of Clinical Sciences, Sahlgrenska Academy at the University of Gothenburg, Sahlgrenska University Hospital, Gothenburg, Sweden; ^2^ Department of Oncology, Institute of Clinical Sciences, Sahlgrenska Academy at the University of Gothenburg, Sahlgrenska University Hospital, Gothenburg, Sweden; ^3^ The Bioinformatics Core Facility at the University of Gothenburg, Gothenburg, Sweden

**Keywords:** melanoma, miR210, hypoxia, xenografts, MEK inhibitor

## Abstract

Cell line-derived xenografts (CDXs) are an integral part of drug efficacy testing during development of new pharmaceuticals against cancer but their accuracy in predicting clinical responses in patients have been debated. Patient-derived xenografts (PDXs) are thought to be more useful for predictive biomarker identification for targeted therapies, including in metastatic melanoma, due to their similarities to human disease. Here, tumor biopsies from fifteen patients and ten widely-used melanoma cell lines were transplanted into immunocompromised mice to generate PDXs and CDXs, respectively. Gene expression profiles generated from the tumors of these PDXs and CDXs clustered into distinct groups, despite similar mutational signatures. Hypoxia-induced gene signatures and overexpression of the hypoxia-regulated miRNA hsa-miR-210 characterized CDXs. Inhibition of hsa-miR-210 with decoys had little phenotypic effect *in vitro* but reduced sensitivity to MEK1/2 inhibition *in vivo*, suggesting down-regulation of this miRNA could result in development of resistance to MEK inhibitors.

## INTRODUCTION

Malignant melanoma arises via stepwise transformation of melanocytes and is highly aggressive when metastatic. Early stage melanomas are often curable by resection, but prognosis and overall survival for patients with advanced-stage malignant melanoma remain poor [1, 2]. The majority of melanomas carry mitogen-activated protein kinase (MAPK) pathway-activating mutations, especially in *BRAF*, *NRAS*, *NF1* [3, 4]; providing avenues for targeted therapeutic intervention. Although clinical responses to targeted therapies are often initially good, most patients eventually develop acquired therapeutic resistance [5]. Likewise, disease-free survival is improved by combination regimens with small molecule inhibitors [6] and/or immunotherapy [7, 8], but most patients succumb to lethal disease. A better understanding of the molecular pathways that govern disease progression and therapeutic resistance is needed to improve clinical outcomes.

Traditional 2-D cell cultures are longstanding and effective models used in basic cancer research. However, cell lines grown in continuous culture adapt to culture conditions, which could contribute to false positive responses to small molecule inhibition *in vitro* and *in vivo* [9, 10]. Patient-derived xenograft (PDX) models are thought to better recapitulate the disease [11, 12]. There is even evidence that drug screens in PDXs could be used to predict treatment responses in patients [13–15]. With advances in next-generation sequencing (NGS) techniques and decreasing prices, it is now accessible to couple these analyses to drug response in PDX models. This makes biomarker discovery possible prior or parallel to clinical trials [16, 17]. The limitation of using PDXs is that these models are not as tractable for genetic and pharmacological high-throughput screens as cultured cells. Hence, cultured cells will continue to contribute to cancer discoveries [18].

The aim of this study was to investigate if gene expression profiles (GEPs) of some widely used metastatic melanoma cell lines could be used to stratify different patient's PDXs - if only the cell lines were grown in mice prior to expression profiling. If this *in vivo-*GEP-normalization would materialize, screening data generated in the cell line *in vitro* could point to which PDX model to use for accurate *in vivo* validation. Instead, our data suggest GEPs from PDXs and CDXs are different, which could be due to a differential regulation of the hypoxic response.

## RESULTS

### Transcriptome-wide differences between CDXs and PDXs with similar somatic mutations

**Figure 1:**
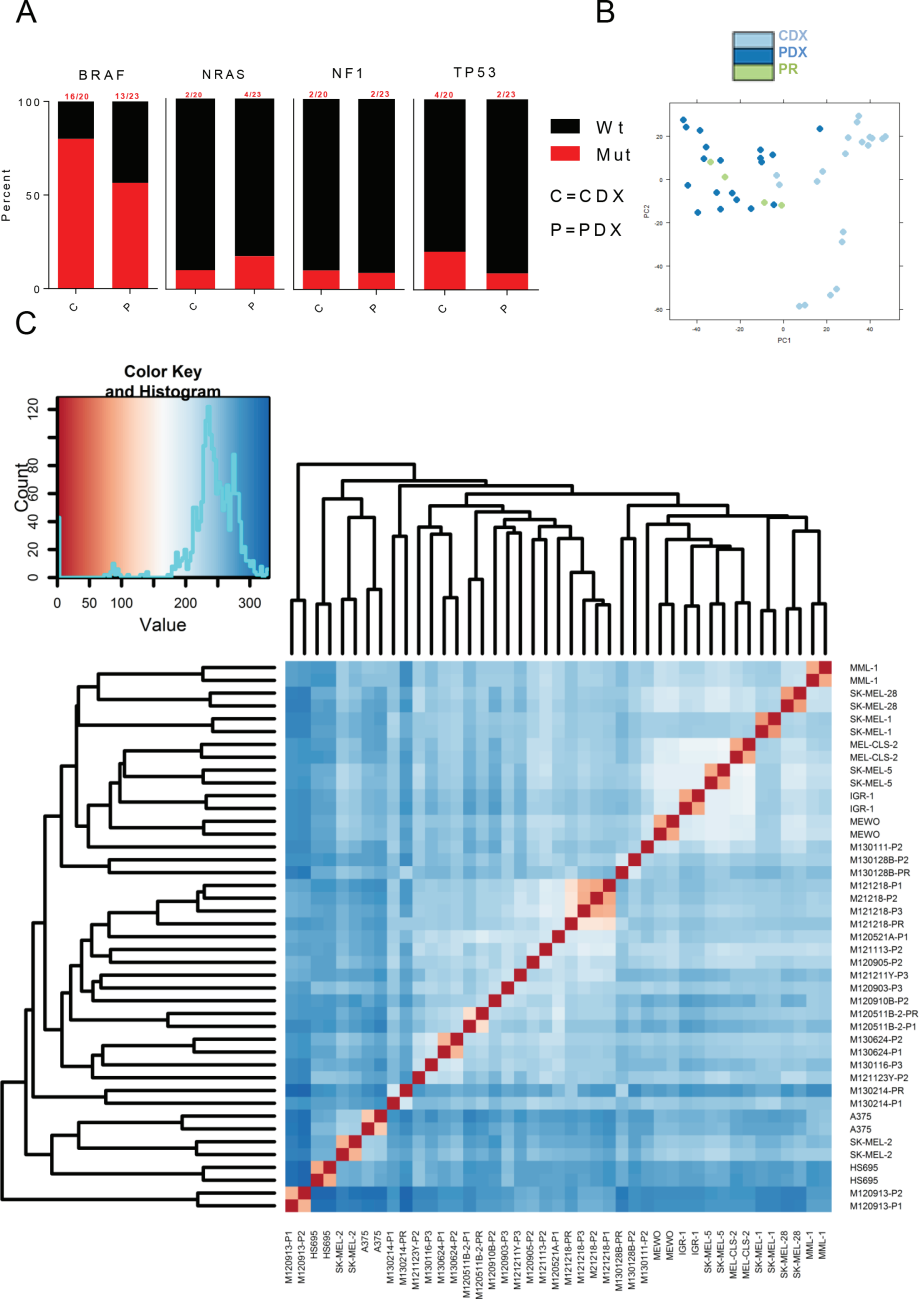
Transcriptome-wide differences between CDXs and PDXs (**A**) Bar graphs showing commonly mutated genes in melanomas across PDXs and CDXs. (**B**) PCA plot of PDXs and CDXs. (**C**) Sample distance matrix of all samples used in the study.

To overcome the problem associated with culture effects on gene expression, we created cell line-derived xenografts (CDXs) by transplanting ten widely used metastatic melanoma cell lines into NOD/Shi-*scid*/IL-2Rγ^null^ (NOG) immunodeficient mice. The CDXs and previously described PDXs and patient tumor biopsies [13] were then analyzed by RNAseq for mutation and transcriptome analyses using poly(A) enrichment. The selected cell line's mutations represented melanoma - eight CDX samples originated from *BRAF* mutant melanoma, SK-MEL-2 harbored an activating Q61R *NRAS* mutation and MeWo carried multiple *NF1* inactivating mutations (Figure 1A and Supplementary Table S1). *TP53* was mutated in SK-MEL-28 (L145R), SK-MEL-2 (G245S) and in two of the PDXs (Supplementary Table S1). Three PDXs harbored no mutations in *BRAF*, *NRAS* or *NF1*.

To assess if the transcriptome of cell lines would be similar to that of PDX or patient biopsies (primary patient biopsies = PR) when cell lines were grown in mice, we performed principle component analysis and sample distance matrix of the transcriptomes of all samples. These analyses revealed that PDX and PR exhibited similar transcriptomes but cell lines appeared different (Figure 1B). This was not due to sample variation since appropriate clustering of the biological duplicates of CDXs and the matching primary or serially transplanted PDX samples was observed (Figure 1C). Notably, the main clusters were not mixed with samples of PDX, biopsies of patient's tumors (PR) and CDX. Instead, PDX and PR were mostly inter-mixed and separate from CDX.

Unsupervised hierarchical clustering of the top-8000 expressed genes in all samples (Supplementary Figure S1A) also show the apparent difference between the transcriptomes of CDX and PDX/PR. It thus appears that PDX and PR are more similar to each other than to CDX.

### Differential regulation of miRNAs between PDXs and CDXs

**Figure 2:**
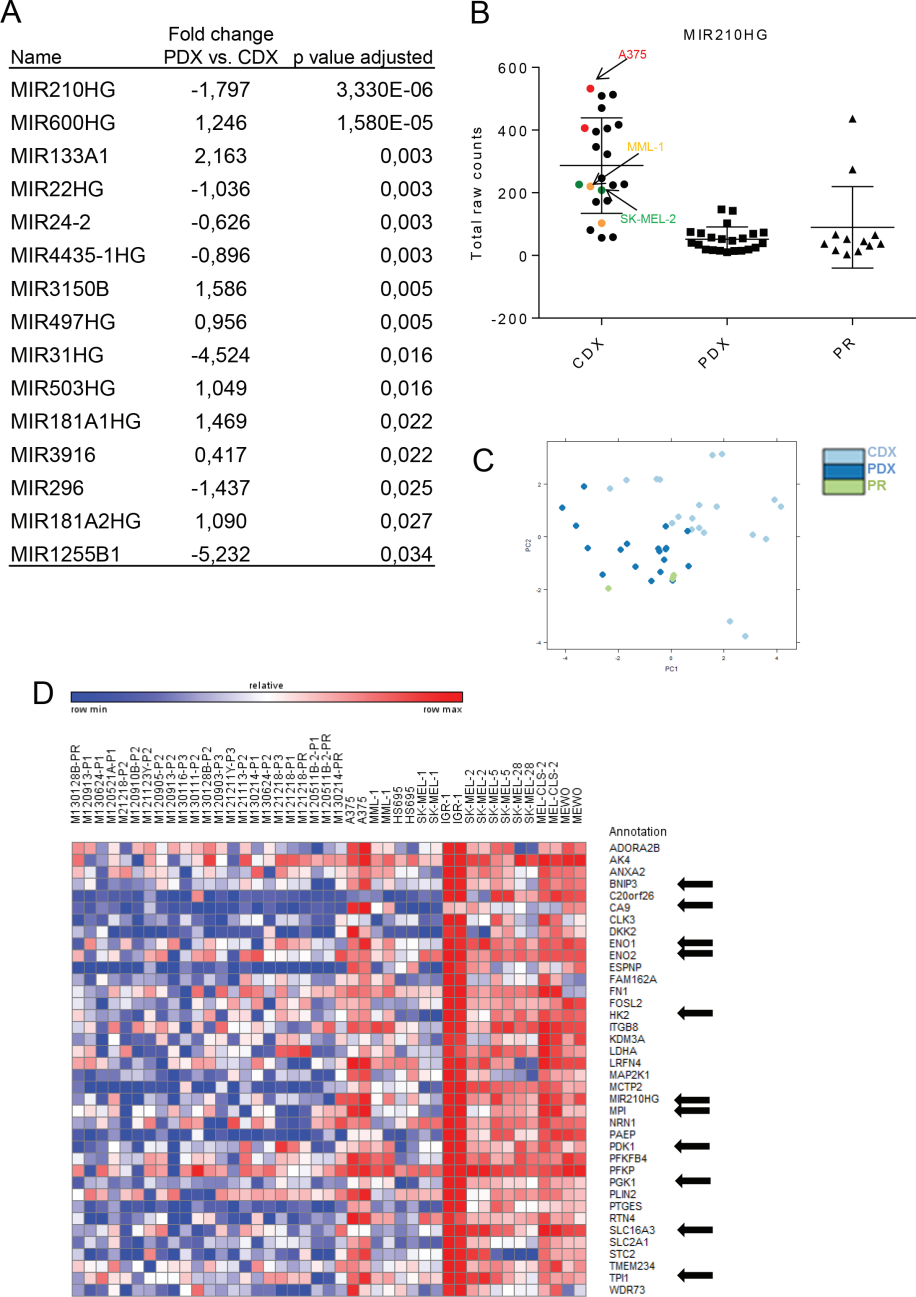
Differential regulation of miRNAs between PDXs and CDXs (**A**) Table showing significantly differentially expressed miRNA's and miRNAHG's between the PDX and CDX. (**B**) Graph showing raw read counts of miR-210HG between Primary, PDX and CDX.(**C**) PCA plot of PDX and CDX. (**D**) Pearson correlation analysis showing top 40 genes that correlated with mir210HG expression.

Micro-RNAs (miRNAs) are small ~21 nucleotide sequences that post-transcriptionally or post-translationally regulate gene expression. It is estimated that at least 30% of all protein-coding genes are regulated by miRNAs in mammals [19] and miRNA regulation is indispensable for mammalian development. miRNAs are known to be dysregulated in cancer and a number of miRNAs have been investigated for their role in melanoma development or therapeutic resistance [20]. Given the clear transcriptome-wide differences between PDXs and CDXs, we next investigated the role of miRNAs by assessing the expression of their host gene mRNAs. Due to their overall low levels of expression, raw reads counts were first extracted from the dataset using “MIR” as the identifier for subsequent differential expression analysis. As with the whole transcriptome data, there were clear differences in miRNA host gene expression between PDXs and CDXs (Figure 2A and Supplementary Table S3). The most significantly altered miRNA host gene between PDXs and CDXs were hsa-miR-210HG and hsa-miR-600HG (Figure 2A–2B and Supplementary Table S3). Moreover, principal component analysis (PCA) (Figure 2C) also revealed differences between PDXs and CDXs, albeit less pronounced than the PCA using the entire transcriptome (Figure 1B). hsa-miR-210HG is a non-protein coding transcript encoding the intronic miRNA hsa-miR-210 whereas no publications or database predictions of function exist for hsa-miR-600HG. Moreover, hsa-miR-210HG contains a hypoxia inducible factor (HIF) response element (HRE), and the non-protein coding transcript is processed to form the mature hsa-miR-210 [21, 22]. Intrigued by the hypoxia regulation, we performed Pearson correlation analysis to identify genes correlating with hsa-mir-210HG. Many of the top 40 genes (marked with an arrow) correlating with hsa-miR210HG expression were either known HIF1 targets and/or involved in glycolysis, as predicted by Gene Set Enrichment Analysis (GSEA) (Figure 2D and Supplementary Table S5). It is thus tempting to speculate that a major difference in transcriptomes between PDXs and CDXs is due to the acclimatization to *in vitro* culture conditions (e.g. 20% oxygen) of the cell lines. When they are then transplanted to mice and experience physiological oxygen tension [23] the cells trigger a pseudo-hypoxic response.

### Abrogation of hsa-miR-210 regulation with a miRNA decoy completely reverses the hypoxia-induced protein expression changes

**Figure 3:**
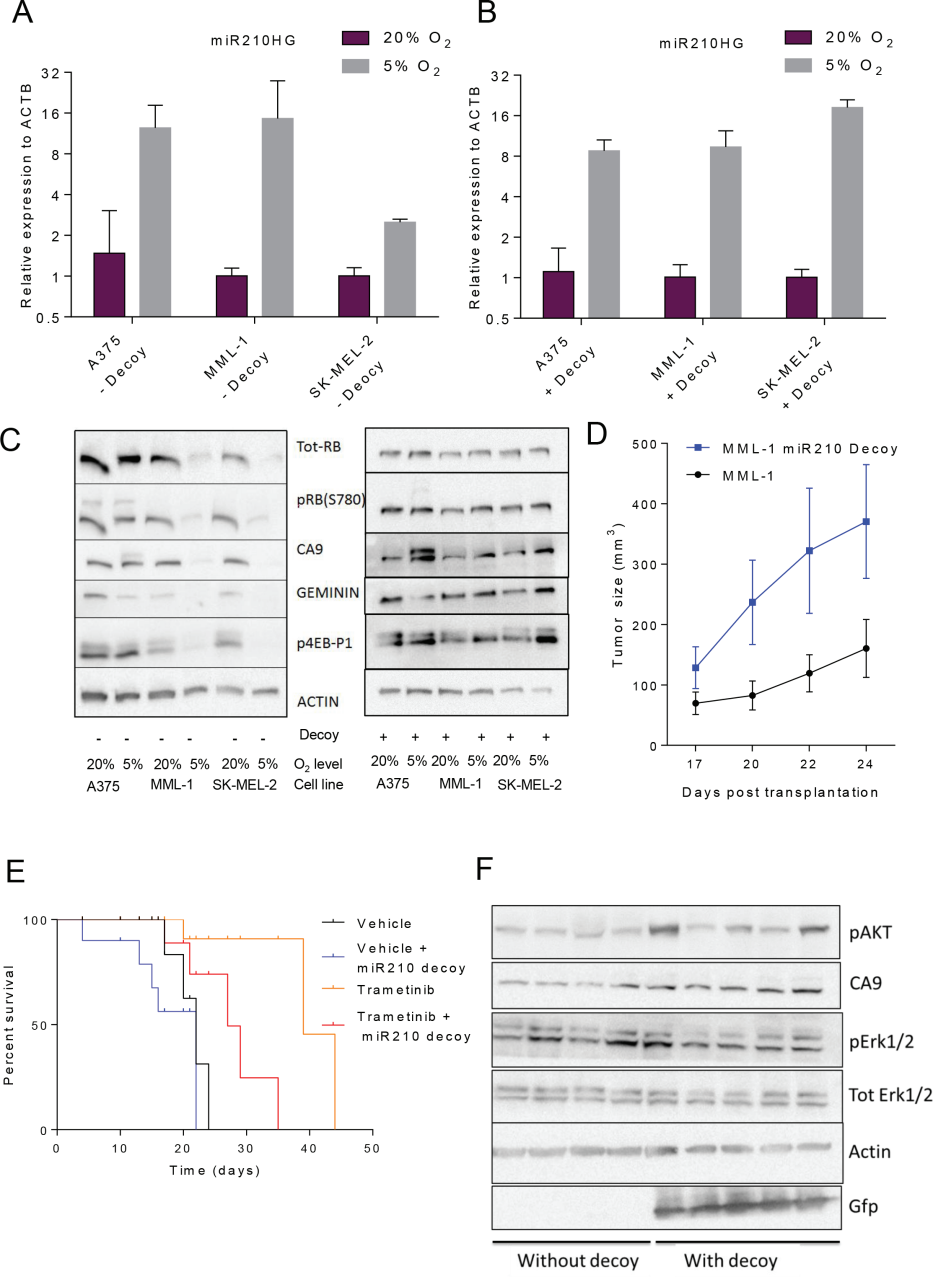
Abrogation of hsa-miR-210 regulation with miRNA decoys completely reverses hypoxia-induced protein translation *in vitro* and makes MML-1 cells less sensitive to MEK inhibition (GSK1120212/trametinib) *in vivo* by accelerating tumor progression (**A**) qRT-PCR analysis shows marked induction of hsa-miR-210HG expression across the three cell lines in response to hypoxia. (**B**) qRT-PCR analysis showing hsa-miR-210HG induction in response to hypoxia across three cell lines engineered with the hsa-miR-210 decoy. (**C**) Western blot analysis of the original and miR210 decoy engineered cell lines under normoxic and hypoxic conditions after 24 h. (**D**) Tumor sizes post transplantation. (**E**) Kaplan-Meier plot showing survival statistics of mice carrying MML-1 wild type or decoy-engineered cells treated with either trametinib or vehicle food. (**F**) Western blot analysis of protein extracts from tumor pieces from MML-1 wildtype or decoy-engineered cells treated with vehicle food only.

Since the transcriptomic analysis suggested that hypoxia was most likely the driver of expression differences between the PDXs and CDXs, we next investigated the role of hsa-miR-210 in the hypoxia-induced phenotype. Three cell lines (A375, MML-1, and SK-MEL-2) were chosen for further experiments, since they had the highest, intermediate, and lowest hsa-miR-210HG read counts in the RNAseq data (Figure 2B and Supplementary Table S4), respectively. Subjecting the cell lines to reduced oxygen levels (5%) for 24 hours induced hsa-miR-210HG expression compared to their respective controls grown in 20% oxygen (Figure 3A). qRT-PCR of selected genes (Supplementary Figure S2) confirmed induction of gene expression in response to hypoxia (Figure 2D, marked with arrow).

We next examined expression of proteins involved in the response of cells to hypoxia. These included phosphorylated RB (p-RB; S780) [24] and geminin [25] as markers of G1 and S-G2M phase of cell cycle, and carbonic anhydrase IX (CA9) which is the most well established target of hypoxia. As expected, p-RB was reduced but so was total RB in MML-1 and SK-MEL-2. One of many possible explanations for reduced total RB could be reduced cap-dependent translation in response to hypoxia [26]. Indeed phosphorylated translation factor 4E-BP1 [27], a marker of cap-dependent translation, was reduced. Contrary to expectation, we did not observe an up-regulation of CA9 in either MML-1 or SK-MEL-2 cell lines grown in 5% oxygen conditions, despite increase in mRNA levels. There was however an increase in a slower migrating band in A375 cells; the cell line with the strongest induction of CA9 mRNA (Figure 3C and Supplementary Figure S2). On the other hand, A375 did not induce the mRNA of many of the other HIF target genes (Supplementary Figure S2), at variance with MML-1 and SK-MEL-2. Taken together these data showed that different cell lines respond differently to lower oxygen and that high hsa-miR-210HG expression correlate with a blocked hypoxia response (except for CA9).

To further investigate the role of hsa-miR-210 in this setting, all the three cells lines were genetically engineered with hsa-miR-210 decoy and subjected to 5% oxygen. Decoys were chosen because they provide prolonged suppression of endogenous miRNA activity compared to hairpin-based RNA inhibition systems [28]. hsa-miR-210HG induction by low oxygen was not hindered by expression of decoy but there the induction of hsa-miR-210HG was stronger in SK-MEL-2, similar in MML-1, and reduced in A375 cells as compared with the same cell lines without the decoy, respectively (Figure 3A and 3B). MML-1 and SK-MEL-2 cells engineered with the miR decoy did not down-regulate the expression of RB and CA9 or loose phosphorylation of 4EBP1 when cultured at 5% oxygen (Figure 3C), suggesting hypoxia response was impaired when these cells were expressing miR decoy. On the hand, some of the hypoxia induced genes such as *BNIP3*, *ENO2* and *SLC16A3* were more robustly induced by 5% oxygen in SK-MEL-2 and MML-1 cells expressing decoy, whereas some genes like *CA9* were less induced (compare Supplementary Figures S2 and S3). It thus appears as if the hypoxia-regulation of translation is impaired but not all of the hypoxic transcriptional regulation in these cell lines. Contrary, A375 cells, were still able to induce a slower migrating form of CA9 when expressing miR decoy (Figure 3C) but the expression levels of the HIF target genes was altered in a similar way as in MML-1 and SK-MEL-2 cells (compare Supplementary Figures S2 and S3). Taken together, these data indicate that the behavior of cells when exposed to low oxygen vary depending on levels of basal hsa-miR-210 expression.

### Loss of hsa-miR-210 function with a miR decoy renders MML-1 cells less sensitive to MEK inhibition (GSK1120212, trametinib) *in vivo* and accelerates tumor progression

Our RNAseq data were generated from melanoma cells grown in mice. Next, we therefore injected MML-1 cells or MML-1 cells engineered with the hsa-miR-210 decoy subcutaneously into the flanks of NOG mice. Decoy expressing MML-1 cells grew more rapidly than ordinary MML-1 cells (Figure 3D) and reached the ethics limit requiring their sacrifice earlier (Figure 3E). To investigate if inhibition of hsa-miR-210 would impact the cells response to targeted therapies, we treated mice with MEK inhibitor trametinib, since this is an approved treatment against BRAF mutated melanoma [29]. As expected, trametinib prolonged life of MML-1 CDXs (Figure 3E). Again, in spite of treatment, mice carrying decoy-engineered MML-1 cells reach the ethics limit for sacrifice significantly faster than mice carrying ordinary MML-1 cells.

To investigate the underlying cause of the relative insensitivity of MML-1 cells expressing decoy, a Reactome Pathway Database map of potential hsa-miR-210 (Supplementary Figure S1B) targets was investigated. Interestingly, both ERK1/2 and AKT, proteins whose induction could bypass MEK inhibition were among the putative targets. Therefore, western blot analysis was performed on protein extracts from tumors arising from MML-1 wild type controls or decoy-engineered cells. There were no marked differences in total or phosphorylated ERK1/2 between the groups, but 3/5 decoy-engineered tumors had increased phosphorylated AKT levels as compared to tumors developed from the parental cell line (Figure 3F).

## DISCUSSION

Cell lines have been used for many decades to study cancer biology and they are believed to capture the genetic variation of human cancer [18]. Moreover, they are generally useful for drug screenings and for genetic high-throughput screening using e.g. RNAi or CRISPR/Cas9. We therefore attempted to generate cell lines to complement our existing PDX biobank [13] but our efforts were mostly unsuccessful. This has been a common issue; generating cell lines from most solid tumors, has been historically difficult [30–34]. To circumvent this we decided to investigate if we could find any similarities between well-known melanoma cell lines and our PDXs. In order to avoid the confounding factor of culture, e.g. fetal bovine serum induced gene expression, we first transplanted all the cell line into mice and generated GEPs from similarly sized tumors as those harvested from PDXs. Unfortunately, we could not generate convincingly inter-mixed clusters of gene expression. GEPs in cell lines were very different from that in patient tumors or in their corresponding PDXs.

There can be a number of reasons why CDXs and PDXs differ in gene expression. When the cell lines were generated, they had to adapt to culture condition that is very different from the physiological conditions. First, growing on plastic could exert a stress for the cancer cell since it normally makes contact to an extracellular matrix. Indeed, 3D cell cultures are viewed as better predictors of therapy responses *in vivo* than 2D cultures [35]. Second, cultured cells do not receive accurate balances of growth factors from fetal bovine serum [36]; some factors can be activating whilst others could be inhibiting growth. Third, cultured cells are generally hyper-glycemic (> 0.2 g/l) in the sense that they are cultured in up to 4.5 g/l of glucose. This high glucose levels could result in excess production of waste products such as lactate, which is not removed by a circulation system as in a tumor tissue. The so-called Warburg effect [37] could hence be a cell culture artifact. Fourth, cells in culture are grown in the presence of 20% oxygen, which could cause oxidative stress and an irreversibly altered cell phenotype [38, 39]. All of these factors likely contribute to selection barriers that are circumvented in a clonal manner. The resulting cell line will be highly adapted to the new conditions and may be very different from a malignant cell residing in a tumor.

In light of our results and the known culture adaptions of cell lines it is tempting to speculate that cell lines transplanted into mice may experience stress due to the physiological growth factor availabilities, oxygen tension and glucose levels, which are all lower than in cell culture. They could respond to this by inducing a hypoxia response including expression of known hypoxia inducible factor regulated genes such as carbonic anhydrase IX (CA9) [40]. Indeed, there was markedly higher expression of *CA9* in CDXs as compared to PDXs and patient biopsies, supporting the notion that the cultured cells became ‘pseudo-hypoxic’ when grown *in vivo*. At this time, we cannot rule out that different growth rates between PDXs and CDXs play a role in the hypoxic appearance of CDXs. We did note however that there was significant variability in growth rates in both groups of xenografts, suggesting that growth condition (normoxia) rather than growth rate dictates the ‘hypoxic’ gene signature. Interestingly, many studies have shown that dysregulated CA9 expression is associated with poor clinical prognosis, the metastatic phenotype, and drug resistance in different cancers [40, 41]. These findings suggest that prolonged cell culture *in vitro* rewires the transcriptome and this could have large consequences for the utility of drug treatment in CDXs as predictors of therapy responses in patients. Reassuringly, and consistent with the observed difference in *CA9* expression, hsa-miR-210HG expression was significantly different between PDXs and CDXs. The role of miRNAs in hypoxia has previously been examined [42], with a number of miRNAs thought to play a role in hypoxia via different mechanisms [43]. hsa-miR-210 is known as the “hypoxiamir” or “hypoxia master regulator” [43], underscoring its role in hypoxia and its regulation. A number of correlations between hsa-mir-210HG expression and hypoxia-related genes known to be regulated by HIF1 or involved in glycolysis supported this here. These data demonstrates that hypoxia contributes to the differences between PDXs and CDXs.

The most surprising data from this study was that all cell lines did not: 1) express similar amount of hsa-miR-210, 2) respond similar to hypoxia in terms of protein expression of CA9, RB or p-4EBP1 and 3) respond similar to expression of hsa-miR-210 decoy [44]. Our results suggest that hsa-miR-210 has a different role in different contexts or cell types. Previously, hsa-miR-210 expression was shown to be associated with aggressive tumors and poor prognosis across malignancies; thereby making it and/or its downstream targets attractive candidates for therapeutic intervention [45–47]. Contrary to that, here we show that engineering MML-1 cells to express a hsa-miR-210 decoy did not block tumor growth *in vivo*, rather it accelerated growth and caused the cells to be less sensitive to inhibition of the established therapeutic target MEK. An obvious difference could be that in our system, we blocked hsa-miR-210 function in the tumor cells only and the cells were grown in an immune compromised host. It is thus plausible that the tumor promoting effects seen e.g. in myeloid-derived suppressor cells expressing hsa-miR-210 [47] is not visible in our system. Hence, our data suggest that hsa-miR-210 belongs to the list of miRNA's including hsa-miR-26 that can act either as an oncogene or as a tumor suppressor [48–50] depending on the cell and the micro-environmental context.

The functional relevance of miRNAs is dependent on the targets that they bind. In the case of hsa-miR-210, we have not established which target(s) is responsible for the reversal of cap-dependent translation inhibition of RB, CA9, aggressive growth *in vivo*, and the reduced sensitivity to MEK inhibitor in MML-1 cells engineered with hsa-miR-210 miR decoy. After mining potential miR targets, we speculated that the decoy would increase total and phospho-ERK1/2. However, there was little difference in phosphorylated (T202/Y204) and total ERK1/2 between groups. There were marginally higher levels of phosphorylated AKT (T308) in some of the tumors arising in the decoy-engineered group. The PI3K/AKT pathways is known to contribute to malignant progression, either via membrane translocation or phosphorylation of AKT [51]. CAP-dependent translation is one of the first processes affected by stress such as hypoxia [27, 52]. It prompts us to hypothesize that the reversal of protein expression in decoy-engineered cells might occur as a result of increased AKT levels, which in turn overrides the protein synthesis machinery [53] and contributes to cellular survival [54], thereby relieving the control mechanism enforced by the miRNA.

To summarize, we have demonstrated that there are distinct transcriptome-wide differences between CDXs and PDXs, likely owing to the adaptions the cell lines once had to undergo to become possible to culture. Since PDXs are transcriptionally similar to the patient biopsies, our data support the undergoing endeavors to use these models by drug developers [16]. Moreover, we show that targeting of hsa-miR-210 in metastatic melanoma cells might have lethal consequences, as they tend to become more aggressive and less sensitive to MEK inhibition *in vivo*.

## MATERIALS AND METHODS

### Mouse strain, xenografts, and treatment

All animal experiments were performed in accordance with E.U. directive 2010/63 (regional Gothenburg animal ethics committee approval #287/289–12 and #36–2014). For cell line xenografts, cells were suspended in RPMI, mixed 1:1 with Matrigel (BD Biosciences), and 2 × 10^5^ cells transplanted subcutaneously into the flanks of immunocompromised, non-obese severe combined immune deficient interleukin-2 chain receptor γ knockout mice (NOG mice; Taconic, Denmark). For transcriptome analyses, all CDX tumors were harvested once they reached 100 −200 mm^3^ in size. To assess effects of trametinib, xenografts whose growth were increased on two consecutive measurements, were randomized to two treatment groups: vehicle or trametinib (4 mg compound/kg food = 1 mg/kg/day) (Selleckchem, Houston, TX) mixed in the fodder (ResearchDiets Inc., New Brunswick, NJ). Mice were weighed and tumors measured using calipers twice a week. Mice were euthanized when tumors were larger than 10 × 10 mm^2^ as per ethical regulations [55].

### Cell culture

All cell lines except HEK-293T (ATCC, Manassas, VA) were purchased from CLS Cell Lines Service GmbH (Eppelehim, Germany) and cultured as per the manufacturer's protocol using media from Gibco (Thermo Fisher Scientific, Waltham, MA) and FBS from HyCLONE (GE Healthcare Life Sciences, USA). All media were supplemented with gentamicin (Thermo Fisher Scientific, Waltham, MA).

### RNA extraction and analysis

RNA was extracted from PDXs and CDXs as described previously [13]. For *in vitro* studies, Quick-RNA™ MiniPrep (Zymo Research, Irvine, CA) was used as per the manufacturer's protocol. cDNA preparation and qRT-PCR were performed as previously described [56] using KiCqStart^®^ SYBR^®^ Green Primers (Sigma-Aldrich, St Louis, MI). Gene expression profiling was performed using RNA sequencing (RNAseq).

### Bioinformatics analysis

To assess purity of the samples, clean reads were mapped to several different species using default settings in PRINSEQ [57]. In none of the samples there was more than 5% reads from mouse, suggesting that contaminating stroma will play a very limited contribution to the gene expression profile. Thereafter, all RNAseq data was analyzed as described previously [13]. The data was annotated using GENCODE v19 [55], and the reads were counted with HTSeq using default parameters. Differential expression analysis for coding and miRNA genes was performed using DESeq2. After normalization, data was transformed by variance-stabilizing transformation. Heatmaps and PCA plots were generated with default DESeq2 functions or using GENE-E (http://www.broadinstitute.org/cancer/software/GENE-E/index.html). Inclusion of genes in heatmaps depended on their *p*-value and/or other analysis as mentioned.

### Virus production and transduction

pCMV-dR8.2 dvpr (plasmid #8455), pMD2.G (a kind gift from Didier Trono (plasmid #12259), and AB.pCCL.sin.cPPT.U6.miR-210-decoy.hPGK.GFP.WPRE (a kind gift from Brian Brown (plasmid # 6599) [44] were purchased from Addgene (Cambridge, MA). Plasmids were isolated using Qiagen Plasmid Plus Midi Kit (Qiagen, Germany) as per the manufacturer's instructions. HEK-293T cells were seeded on 6 cm plates (Sarsted, Germany) one day prior to transfection. Calcium phosphate-mediated transfection was performed using packaging:envelope:transfer plasmids at a ratio of 0.259:0.087:0.345 μg/cm^2^, and virus was harvested 42, 46, 50 and 66 hours post transfection. The virus containing supernatant passed through 0.45μM low protein-binding filter (Sarstedt, Germany) filters.

A375, MML-1, and SK-MEL-2 cells were transduced with 1 ml of virus containing 2–4 μg/ml of polybrene (Sigma-Aldrich) per cell line and seeded on 6-well plates (Sarstedt, Germany). 12 h post transduction, fresh media was added and cells were allowed to grow for another 24 h. All cell lines were analyzed with BD Accuri C6 FCM (BD Biosciences, USA) for GFP expression before further experiments were performed (data not shown).

### Immunoblotting

Cells were lysed as previously described [56]. Protein extracts (20–45 μg per lane) were separated by electrophoresis on ClearPAGE (C.B.S. Scientific, San Diego, CA) and transferred to 0.22 μM nitrocellulose membranes (Protran; GE Healthcare Bio-Sciences). Ponceau-S (0.2% solution; Serva Electrophoresis, Heidelberg, Germany) staining was performed following transfer to confirm successful transfer. Post transfer, membranes were blocked with 5% BSA (Wt/Vol) in TBST (Santa Cruz Biotechnology, USA) for 1 h at room temperature followed by probing with specific antibodies (Supplementary Table S3) overnight at 4–8°C. Appropriate Amersham ECL HRP-conjugated antibodies (GE Healthcare Bio-Sciences, USA) were used as secondaries. Membranes were developed using Luminata Forte ECL substrate (EMD Millipore, Billerica, MA) and imaged with the LAS-1000 imager (Fuji Films, Japan).

## SUPPLEMENTARY FIGURES AND TABLES












