# Supplementary material for: Hypoxia-regulated gene expression explains differences between melanoma cell line-derived xenografts and patient-derived xenografts
Source: Oncotarget. 2016 Mar 18;7(17):23801–11. doi: 10.18632/oncotarget.8181 (PMC5029664; doi:10.18632/oncotarget.8181)
Supplement: Supplementary file 1 [file oncotarget-07-23801-s001.pdf]

Hypoxia-regulated gene expression explains differences between melanoma cell line-derived xenografts and patient-derived xenografts

Supplementary Materials

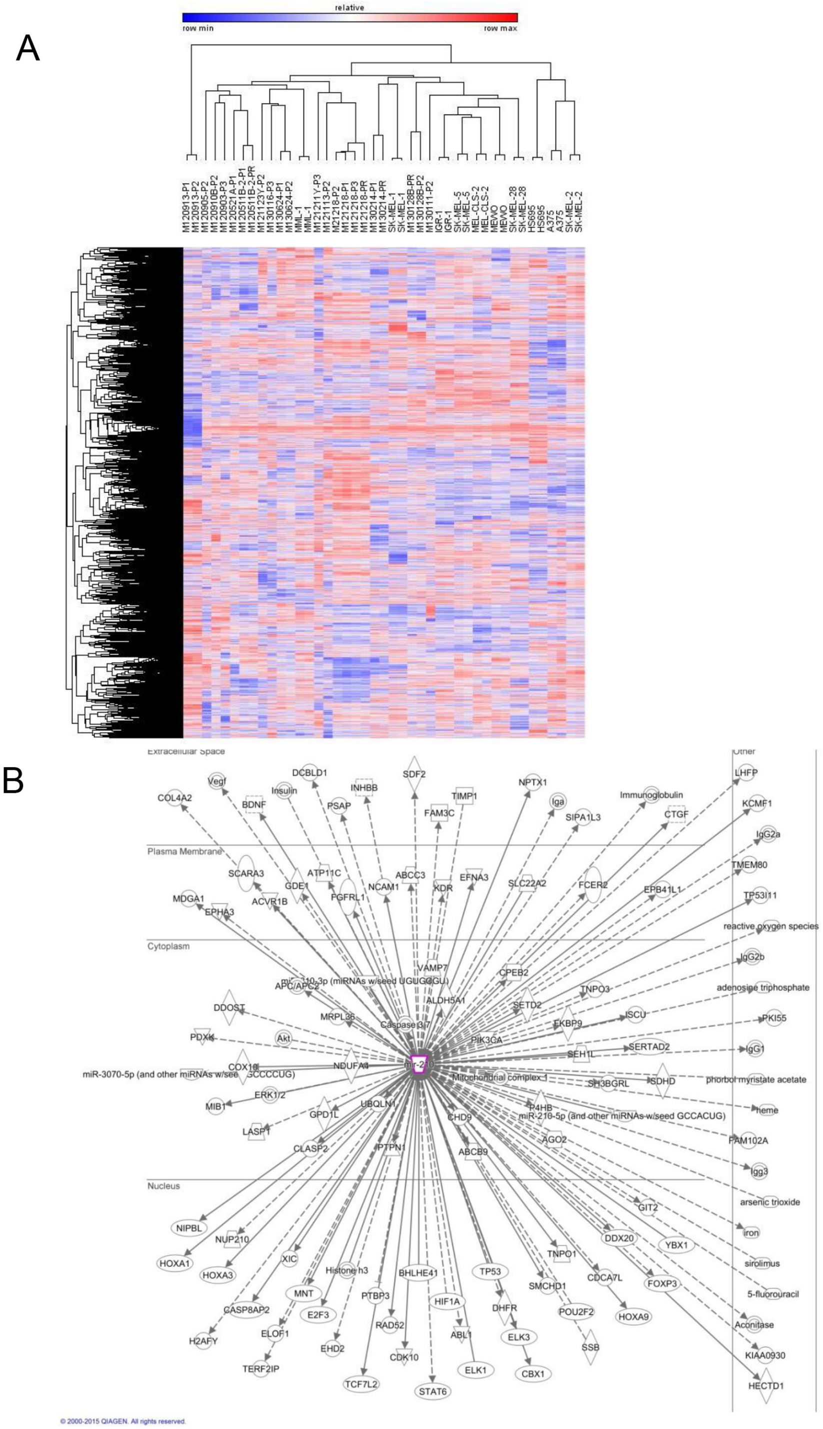

Supplementary Figure S1: (A) Hierarchical clustering of top 8000 genes using Eucledian distance. (**B**) hsa-miR-210 reactome map as generated using IPA (Qiagen, Germany) showing validated targets.

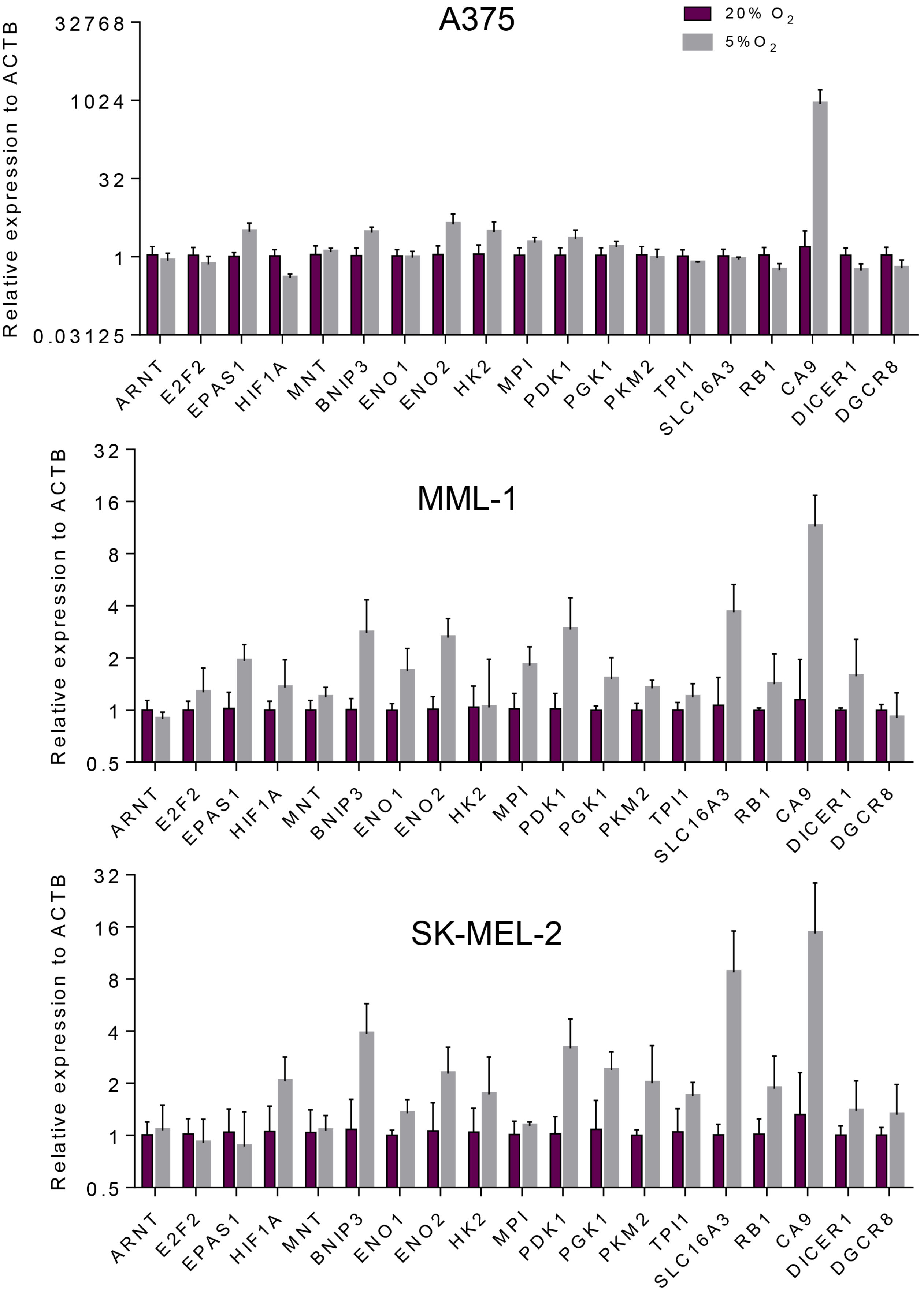

Supplementary Figure S2: qRT-PCR analysis of select genes in wild type A375, MML-1 and SK-MEL-2 cell lines after exposure to 20% and 5% oxygen levels for 24 hours.

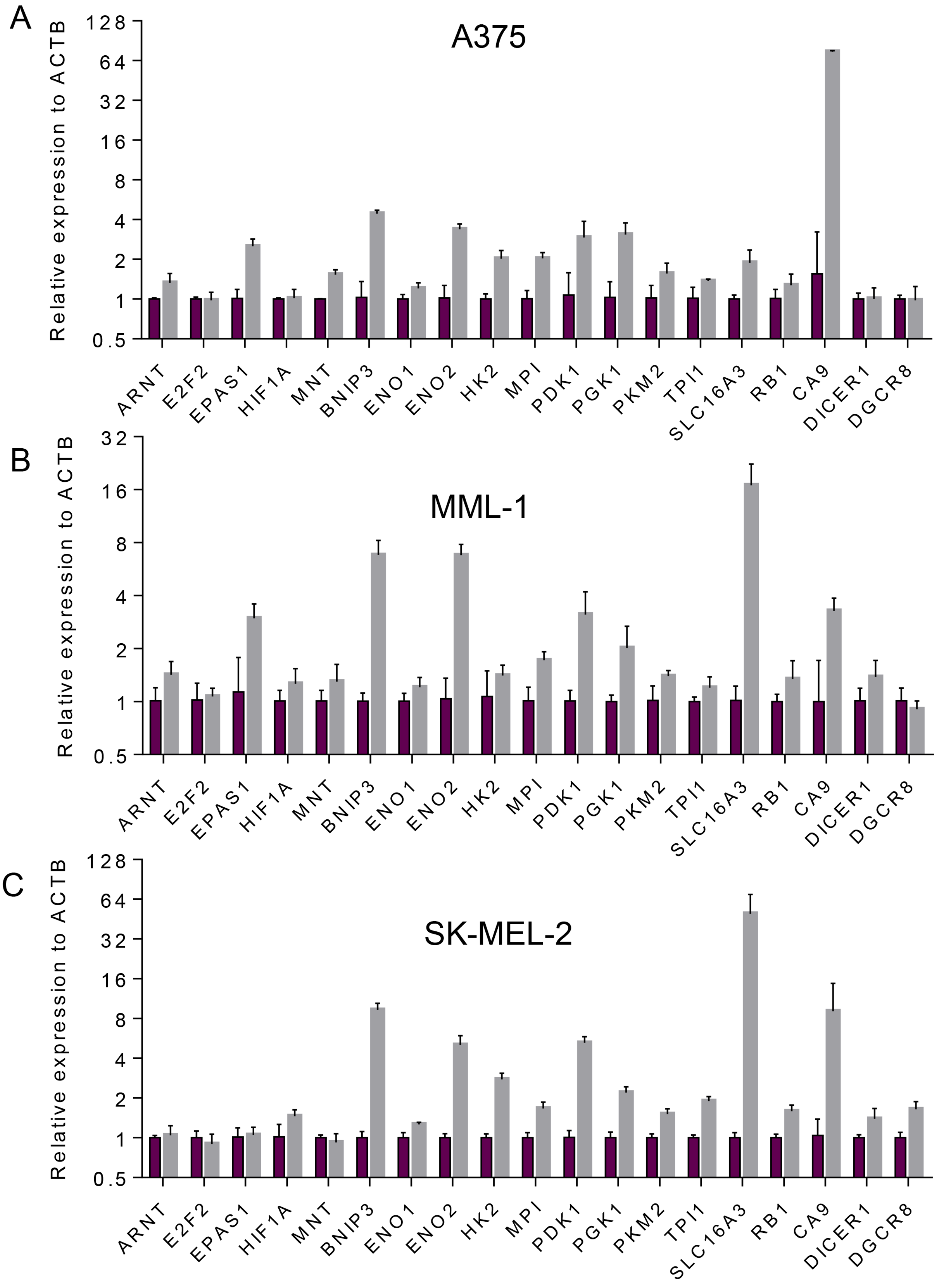

Supplementary Figure S3: qRT-PCR analysis of select genes in A375, MML-1 and SK-MEL-2 cell lines engineered with mir210 decoy after exposure to 20% and 5% oxygen levels for 24 hours.

Supplementary Table S1: Mutational status of the commonly mutated genes in melanoma across the samples CDX= Cell line derived xenograft; PR= Primary patient biopsy; PDX= Patient derived xenograft

Supplementary Table S2: Differential expression of all genes between PDX vs CDX, PR VS PDX and PR vs CDX. Data sorted by pADJ

Supplementary Table S3: Differential expression of miRNA and miRNA host genes (miR-HG) between PDX vs CDX, PR VS PDX and PR vs CDX. Data sorted by pADJ

Supplementary Table S4: Varriance stablised data expression level of all genes annotated across the samples and sorted using highest median expression level of a gene across the samples

Supplementary Table S5: Overlap results from GSEA (http://www.broadinstitute.org/gsea/index.jsp)

Supplementary Table S6: Antibody used

Target	Catalouge Number	Company	Dilution used
pRB (S780)	9307	Cell Signaling	1:1000
Total RB	G3-245	BD Biosciences	1:1000
Total Erk1/2	4695	Cell Signaling	1:1000
pErk1/2 (T202/Y204)	4370	Cell Signaling	1:1000
pAKT (T308)	13038	Cell Signaling	1:1000
CA9	ab15086	AbCAM	1:1000
GEMININ (FL-209)	sc-13015	SBCT	1:1000
p4E-BP1 (T37/46)	2855	Cell Signaling	1:1000
GFP	632380	Takara	1:4000
β-Actin	AC-15/A1978	Sigma-Aldrich	1:10000
α-Rabbit	NA934	GE Healthcare lifesciences	1:5000
α-Mouse	NA931	GE Healthcare lifesciences	1:5000
